# SARS CoV‐2 related microvascular damage and symptoms during and after COVID‐19: Consequences of capillary transit‐time changes, tissue hypoxia and inflammation

**DOI:** 10.14814/phy2.14726

**Published:** 2021-02-01

**Authors:** Leif Østergaard

**Affiliations:** ^1^ Neuroradiology Research Unit Section of Neuroradiology Department of Radiology Aarhus University Hospital Aarhus Denmark; ^2^ Center of Functionally Integrative Neuroscience Department of Clinical Medicine Aarhus University Aarhus Denmark

**Keywords:** brain, capillary dysfunction, COVID‐19, heart, hypoxemia, hypoxia, inflammation, long‐term symptoms, lungs, microcirculation, muscle

## Abstract

Corona virus disease 2019 (COVID‐19) causes symptoms from multiple organs after infection by severe acute respiratory syndrome corona virus 2 (SARS CoV‐2). They range from early, low blood oxygen levels (hypoxemia) without breathlessness (“silent hypoxia”), delirium, rashes, and loss of smell (anosmia), to persisting chest pain, muscle weakness and ‐pain, fatigue, confusion, memory problems and difficulty to concentrate (“brain fog”), mood changes, and unexpected onset of hypertension or diabetes. SARS CoV‐2 affects the microcirculation, causing endothelial cell swelling and damage (endotheliitis), microscopic blood clots (microthrombosis), capillary congestion, and damage to pericytes that are integral to capillary integrity and barrier function, tissue repair (angiogenesis), and scar formation. Similar to other instances of critical illness, COVID‐19 is also associated with elevated cytokine levels in the systemic circulation. This review examines how capillary damage and inflammation may contribute to these acute and persisting COVID‐19 symptoms by interfering with blood and tissue oxygenation and with brain function. Undetectable by current diagnostic methods, capillary flow disturbances limit oxygen diffusion exchange in lungs and tissue and may therefore cause hypoxemia and tissue hypoxia. The review analyzes the combined effects of COVID‐19‐related capillary damage, pre‐existing microvascular changes, and upstream vascular tone on tissue oxygenation in key organs. It identifies a vicious cycle, as infection‐ and hypoxia‐related inflammation cause capillary function to deteriorate, which in turn accelerates hypoxia‐related inflammation and tissue damage. Finally, the review addresses the effects of low oxygen and high cytokine levels in brain tissue on neurotransmitter synthesis and mood. Methods to assess capillary functions in human organs and therapeutic means to protect capillary functions and stimulate capillary bed repair may prove important for the individualized management of COVID‐19 patients and targeted rehabilitation strategies.

## Graphical abstract

COVID‐19‐related microvascular damage and inflammation may cause tissue hypoxia via transit‐time effects and disturb neurotransmitter synthesis in the brain. The duration of COVID‐19 symptoms and the long‐term health effects of SARS‐CoV‐2 infection may rely on whether disease‐related capillary damage is reversible.

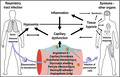

## 1 INTRODUCTION

In the past year, severe acute respiratory syndrome coronavirus 2 (SARS‐CoV‐2) infections have swept across continents, claiming over 1.5 million lives (The Johns Hopkins Coronavirus Resource Center (CRC), 2020). First considered a respiratory disease, coronavirus disease 2019 (COVID‐19) also affects other organ systems, including the brain, heart, kidneys, liver, skeletal muscle, and skin of infected patients.

SARS‐CoV‐2 is asymptomatic in some, whereas others develop severe symptoms, some requiring ventilator treatment. Elderly patients, and patients with preexisting respiratory disease or cardiovascular risk factors, are at greater risk of a severe disease course (Liu et al., 2020). In many patients, symptoms persist after the infection, affecting patients’ return to work and quality‐of‐life—see Table 1 (Yelin et al., 2020). While most symptoms disappear over the weeks and months following the infection, the extent of long‐term COVID‐19 sequela remains unclear.

**TABLE 1:** Long‐term complaints of people recovering from acute COVID‐19. Adapted from Yelin et al. (2020)

Extreme fatigue
Muscle weakness
Low‐grade fever
Inability to concentrate
Memory lapses
Changes in mood
Sleep difficulties
Headaches
Needle pains in arms and legs
Diarrhea and bouts of vomiting
Loss of taste and smell
Sore throat and difficulties to swallow
New onset of diabetes and hypertension
Skin rash
Shortness of breath
Angina (Chest pains)
Palpitations (irregular heartbeat)

SARS‐CoV‐2 infection affects the vascular system and blood's coagulation properties, injuring vascular walls and causing blood clots to form in both large and microscopic blood vessels (Ackermann et al., 2020; Liu et al., 2020; Teuwen et al., 2020). While we are familiar with the pathophysiology, symptoms, diagnostic findings, and management of large vessel occlusions (e.g., acute stroke, coronary occlusion, pulmonary embolism, deep venous thrombosis), the consequences of microcirculatory disturbances, for example, the narrowing or occlusion of individual capillaries, remain unclear (Ackermann et al., 2020; Fox et al., 2020; Varga et al., 2020). This review addresses this knowledge gap (Østergaard, 2020).

Our current vascular disease paradigm focuses on *blood flow*‐*limiting* conditions on the one hand, and symptoms of hypoxia and hypoxic tissue injury, on the other. Blood's capillary transit time, however, determines the time availability for blood‐tissue oxygen exchange, and any deviations from a homogenous distribution of blood across the capillary bed therefore reduce the capillary bed's functional capillary permeability surface area product (PS) for oxygen transport (Jespersen & Østergaard, 2012; Østergaard, 2020). Therefore, blood's uptake of oxygen in the lungs, and the uptake of oxygen by tissue, not only depend on *blood flow*, but also on bloods *microscopic distribution across the capillary bed* (Angleys & Østergaard, 2020; Hsia et al., 1999; Jespersen & Østergaard, 2012; Østergaard, 2020). Biophysically, the redistribution of blood among open capillaries can account for the puzzling increase in oxygen PS observed as blood flow increases through most organs, without the need to invoke classical “capillary recruitment” (the active opening of previously closed capillaries), which has now been ruled out in most organs (Østergaard, 2020; Poole et al., 2020). This review focuses on the effects of *break*‐*downs* in the factors that ensure the crucial, parallel increase in PS and blood flow, after biophysical models have predicted that capillary occlusions and severe capillary flow disturbances can limit tissue oxygenation to the *same extent* as symptomatic reductions in blood flow (ischemia) in brain, heart, and kidneys (Østergaard et al., 2013, 2014, 2015).

Consistent with these predictions, patients with mild cognitive impairment (MCI) and Alzheimer's Disease (AD), who show widespread cerebral microvascular flow disturbances compared to controls (Eskildsen et al., 2017; Nielsen et al., 2020), also display loss of cognitive functions across patients and over time in proportion to these disturbances and the resulting decline in critical brain regions’ ability to extract blood's oxygen (Eskildsen et al., 2017; Nielsen et al., 2017). Such *capillary dysfunction* is believed to develop over decades, as microvascular injuries accumulate due to aging, risk factor, and diseases—but only to cause symptoms when the injuries reach a certain threshold. The review examines how sudden, COVID‐19‐related microvascular changes affect oxygen availability in subjects with different, pre‐existing levels of capillary dysfunction, and asks whether, for example, unexpected hypertension and COVID‐19‐related cognitive symptoms (“brain fog”) are related to transient reductions in blood and brain oxygenation.

Reductions in tissue oxygen levels activate inflammation and cytokine release (Eltzschig & Carmeliet, 2011), which may interfere with neurotransmission, just as oxygen is important for the brain's serotonin synthesis (Østergaard et al., 2018). The review discusses how microvascular damage and inflammation could affect brain functions, including mood.

The availability of oxygen in tissue depends on the function of its capillaries as well as arterial blood's oxygen concentration, both of which may be limited by COVID‐19. This integrative review therefore addresses putative consequences of capillary flow disturbances in both lungs and other tissues.

## 2 HOW CAN COVID‐19‐RELATED CAPILLARY CHANGES AFFECT BLOOD OXYGENATION?

The oxygen uptake in lung alveoli is limited by capillary blood's mean transit time (MTT), the time available for blood‐air oxygen exchange in lung alveoli before blood returns the heart. Capillary occlusions in the lung can therefore limit blood's oxygen uptake as the cardiac output has to pass through fewer capillaries, only faster. In patients who died from respiratory failure caused by SARS‐CoV‐2, Ackermann et al. found alveolar capillary microthrombi to be nine times more prevalent than in patients who died from acute respiratory distress (ARDS) secondary to influenza A[H1N1] (Ackermann et al., 2020), suggesting that alveolar capillary occlusions are characteristic of COVID‐19. Unlike influenza‐related ARDS, COVID‐19 was further characterized by significant new blood vessel formation by intussusceptive angiogenesis, giving rise to substantial distortions of the alveolar capillary plexus’ architecture (Ackermann et al., 2020). The benefits, in terms of blood oxygenation, of this response to parenchymal hypoxia is contingent on alveolar ventilation as well as the function of these new, chaotic microvessels, keeping in mind that angiogenesis tends to result in capillaries that act as “shunts” for blood through the microcirculation (Pries et al., 2010). Reynolds et al. found evidence of blood being shunted through the lung circulation in more COVID‐19 patients with severe pneumonia (83%) than in patients with ARDS (26%; Reynolds et al., 2020a). Unlike ARDS patients, the extent of this shunting was associated with poor blood oxygenation in COVID‐19 patients (Reynolds et al., 2020a). The authors attribute these findings to abnormally dilated alveolar capillaries with diameters in excess of 24 μm (normal alveolar capillaries <15 μm; Reynolds et al., 2020b), consistent with significant capillary shunting, possibly related to changes in alveolar angio‐architecture and congested, parallel capillary pathways.

The alveolar blood–air barrier is less than a micrometer thick and provides efficient gas exchange by its high permeability to oxygen and 5–10 times higher permeability to carbon dioxide (CO_2_). In ARDS, fluid, immune cells, and cell debris accumulate in lung alveoli and within alveolar walls, preventing gas exchange so poorly oxygenated, CO_2_‐rich blood returns to the heart – so‐called *right*‐*to*‐*left shunting*. *Hypoxic vasoconstriction* counteracts such shunting and matches perfusion and ventilation across normal lungs by limiting blood flow through poorly ventilated alveoli.

The apparent capillary transit‐time effects in COVID‐19 differ from “traditional” right‐to‐left shunting in two respects: First, transit time effects limit blood's oxygen uptake even in well‐aerated alveoli. Second, CO_2_ exchange is largely insensitive to transit‐time effects due to its high permeability. In patients with well‐ventilated alveoli, transit‐time effects are therefore expected to cause *normocapnic hypoxemia*, and may therefore contribute to “silent hypoxia” because hypercapnia, in some, confers the sense of breathlessness. Reports of severe hypoxemia in COVID‐19 patients whose lung compliance and chest CT suggest that their alveoli remain both aerated (Gattinoni et al., 2020) and perfused (Lang et al., 2020), are also consistent with severe alveolar capillary microthrombosis and capillary shunting early in the course of the disease.

In patients with obliterative lung diseases, such as emphysema and pulmonary fibrosis, lung capillary blood volume is reduced (Hsia, 2002) and alveolar MTT (~the ratio between the lungs’ capillary blood volume and cardiac output) may therefore become critically short if hypoxic vasoconstriction redistributes blood among fewer alveoli and capillary occlusions further shorten individual alveolar MTT. Transit‐time effects may therefore contribute to the vulnerability of these patients to COVID‐19.

## 3 PRE‐EXISTING CAPILLARY DYSFUNCTION AND OXYGEN AVAILABILITY IN TISSUE

**FIGURE 1:**
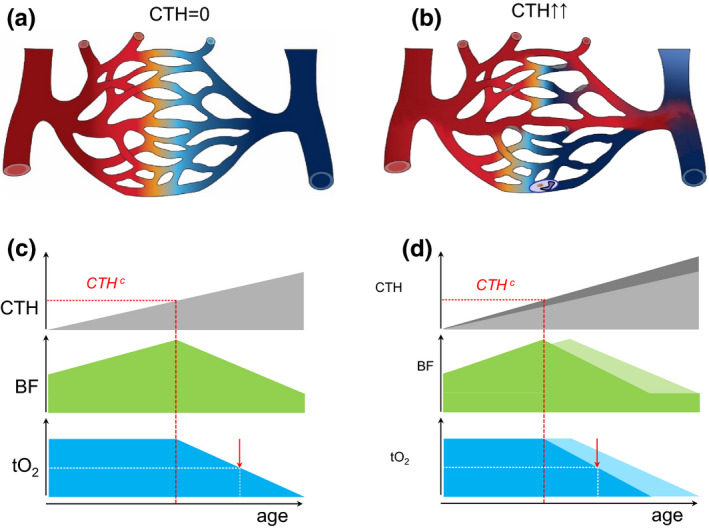
Capillary dysfunction and tissue oxygenation (a) Schematic capillary bed. Fully oxygenated blood is indicated by red and its transition to blue illustrates gradual deoxygenation as oxygen diffuses into tissue. In this illustration, the distribution of blood across parallel capillary paths is homogenous (no capillary transit‐time heterogeneity, i.e., CTH=0), providing optimal oxygen extraction. Modified from (Østergaard, 2020). (b) Capillary flow disturbances can be caused by either a narrowing or a widening of individual capillary segments, or by altered blood properties, such as reduced erythrocyte deformability or neutrophil adhesion after glycocalyx shedding. Note how the inability to homogenize capillary transit times across the capillary bed (CTH > 0) leads to poorer oxygen extraction although blood flow is identical to panel a. Modified from (Østergaard, 2020). (c) This panel illustrates how, for modest increases in CTH, tissue oxygen tension (tO_2_) can be maintained by a compensatory *increase* in blood flow, contrary to our current vascular disease paradigm (Østergaard, 2020). The dashed red line indicates CTH^c^, above which metabolic demands can *only* be met by limiting blood flow (Angleys et al., 2015; Jespersen & Østergaard, 2012). The lower blood supply causes tO_2_ to decrease, whereas the longer blood transit times and higher blood‐tissue oxygen concentration gradients provide more efficient oxygen extraction from blood, understood as a higher oxygen extraction fraction (OEF). The futility of increasing blood flow beyond the capillary bed's capacity to extract blood's oxygen may be reflected in reperfusion injury after ischemic episodes, during which capillaries irreversibly constrict (O'Farrell et al., 2017; Yemisci et al., 2009). As CTH increases beyond CTH^c^, BF, and tO_2_ gradually decreases. The red arrow indicates a 50% reduction in tissue oxygen tension to highlight the threat to critical, ATP‐sensitive cell processes and organ functions. (d) The curves illustrate how cardiovascular risk factors that accelerate capillary injury (Østergaard et al., 2016) modify the curves displayed in panel c (indicated in lighter, gray, green and blue, respectively). Note how CTH^c^ and the onset of critically low tissue oxygen levels are expected to be reached earlier in the affected tissues. The clinical correlates of this earlier attenuation of blood flow may be earlier onset of endothelial dysfunction and hypertension, higher morbidity due to accelerated microvascular injury in critical organs, and lower life expectancy

Transit time effects can limit air‐blood and blood‐tissue oxygen transport even in the *absence* of capillary occlusions. Oxygen extraction from the capillary bed is most efficient if end‐capillary oxygen concentrations are equal (Jespersen & Østergaard, 2012; Lucker et al., 2018) – see Figure 1a. Any capillary flow disturbances, for example, due to endothelial swelling or the slow passage of activated neutrophils through some capillaries, shorten bloods transit times through the remaining, patent capillaries, limiting oxygen uptake (Østergaard, 2020) – see Figure 1b.

*Capillary transit*‐*time heterogeneity* (CTH) quantifies the extent of capillary flow disturbances, typically in terms of the *standard deviation* of capillary transit times or blood velocities within a microscopic tissue volume. Importantly, CTH also quantifies the combined, accumulated impact of aging, risk factors, and disease‐related microvascular damage on capillary hemodynamics at a given time point—and allows its impact on tissue oxygenation to be calculated, alongside that of tissue blood flow, by biophysical models (Angleys et al., 2015; Jespersen & Østergaard, 2012).

Tissue blood flow (BF) is closely regulated to meet local metabolic demands while maintaining tissue oxygen tension (tO_2_) within a narrow range. Figure 1c illustrates how tissue blood flow (BF) can sustain a constant rate of oxygen metabolism as capillary function deteriorates (CTH increases, PS decreases) over time. Notice how a gradual BF *increase* initially compensates for the reduction in oxygen extraction efficacy. Because of the opposing effects of higher blood supply and shorter MTT on tissue oxygenation, however, CTH invariably reaches a critical threshold, CTH^c^, beyond which further vasodilation can no longer sustain normal tO_2_. To meet metabolic demands, BF must instead be *reduced* to limit the “shunting” of oxygenated blood through the microcirculation—at the expense of a gradual reduction of tO_2_. Note that such critical levels of capillary shunting can be reached at any CTH level by a sufficiently large blood flow increase (MTT reduction; Angleys et al., 2015; Jespersen & Østergaard, 2012). The critical transition at CTH^c^ is therefore expected to be presaged by a gradual attenuation of vasodilator responses before this threshold is reached, as the accompanying blood flow increases critically shorten transit times. This property has been linked to *endothelial dysfunction* (attenuated flow responses to endothelial‐dependent vasodilators) characteristic of cardiovascular risk factors, and the subsequent increase in vascular resistance and vascular wall remodeling associated with hypertension (Østergaard, 2020).

As CTH increases further, tissue oxygen tension is expected to fall to levels where critical cell functions become compromised (red arrow), after which failure of critical organ functions may ensue.

Figure 1d illustrates how accelerated deterioration of capillary function due to life‐long exposure to vascular risk factors (e.g., hypercholesterolemia, smoking) is expected to result in earlier onset of capillary dysfunction‐related disorders (Østergaard et al., 2016) and possibly reduce life expectancy.

**FIGURE 2:**
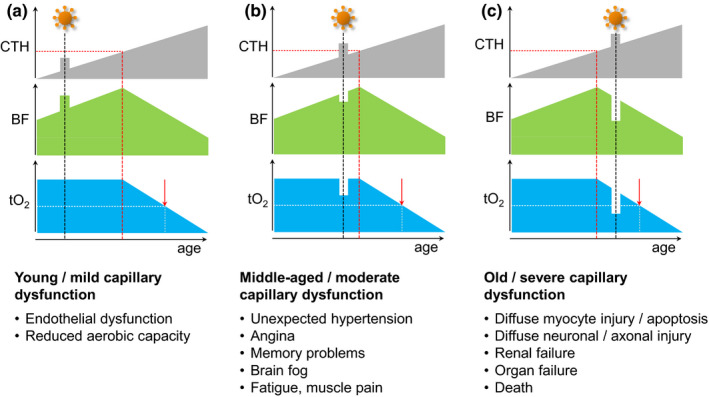
Temporary capillary flow disturbances and tissue oxygenation. This figure illustrates how temporary COVID‐19‐related flow disturbances are expected to affect flood flow (BF) and tissue oxygen tension (tO_2_). Panels show expected responses in tissue with mild (panel a), moderate (panel b), and severe (panel c) pre‐existing capillary dysfunction. (a) If tissue CTH remains below CTH^c^ during COVID‐19‐related capillary flow disturbances, tO_2_, and thereby organ functions during rest, are likely to remain unaltered. For organs that rely on capillary transit time homogenization to meet their metabolic demands during work, such as the heart and skeletal muscle (Angleys & Østergaard, 2020), the additional capillary dysfunction is expected to reduce maximum aerobic capacity, affecting the performance of, for example, young athletes. These signs are expected to reverse upon successful therapeutic or intrinsic capillary recanalization/repair. Irreversible changes to the capillary bed's oxygen extraction capacity, however, may accelerate the emergence of symptoms and disease changes in the affected tissues, cf. Figure 1d. (b) For patients with asymptomatic, moderate preexisting capillary flow disturbances, a COVID‐19‐related CTH increase may cause them to exceed CTH^c^ and lead to a reduction in blood flow and tissue oxygen tension. While, for example, MCI‐like symptoms are expected to disappear with disease‐related capillary flow disturbances, they may herald developing capillary dysfunction and the benefits of managing cardiovascular risk factors to prevent or delay their recurrence in later life (Hachinski et al., 2019). In COVID‐19 patients with pre‐existing myocardial capillary dysfunction, a sudden CTH increases may reduce tO_2_ to levels so that they experience angina during exercise in the absence of flow‐limiting coronary disease (Østergaard et al., 2014). (c) In patients with symptomatic, pre‐existing severe capillary dysfunction, further increases in CTH are expected to cause immediate drops in oxygen availability and worsen preexisting symptoms (e.g., confusion or delirium in patients who already have dementia, myocardial damage, or kidney failure). If oxygen levels fall below the metabolic requirement of critical cell functions, tissue and organ damage is expected to cause lasting symptoms or even organ failure and premature death

Figure 2 illustrates how short‐term COVID‐19‐related capillary flow disturbances (increased CTH) are predicted to affect tissue oxygenation and organ functions for subjects with different levels of pre‐existing age and risk factor‐related capillary dysfunction. In younger subjects, capillary damage is expected to be asymptomatic, except during exercise where lung, heart, or muscle capillary function is affected—Figure 2a. Note how patients with asymptomatic pre‐existing capillary dysfunction in brain tissue (Figure 2b) could experience the sudden deterioration to CTH and tissue oxygen levels characteristic of mild cognitive impairment or AD (Nielsen et al., 2020) as “brain fog” and memory problems. In elderly patients with symptomatic pre‐existing capillary dysfunction in certain organs, additional capillary flow disturbances are expected to worsen symptoms significantly and to potentially be life‐threatening—Figure 2c.

## 4 SARS‐COV‐2 INFECTION AND ITS EFFECT ON THE MICROCIRCULATION

The SARS‐CoV‐2 virus can bind to angiotensin‐converting enzyme 2 (ACE2) receptors on cell surfaces and be internalized by the cell (Liu et al., 2020). The virus‐receptor interaction is thought to reduce ACE2 action and increase levels of angiotensin II, a powerful capillary and arteriolar vasoconstrictor (Capone et al., 2011; Kawamura et al., 2004; Tilton et al., 1979) that propagates thrombogenicity, oxidative stress, and inflammation (Liu et al., 2020). With respect to COVID‐19 respiratory symptoms, alveolar epithelial, and capillary endothelial cells express ACE2 receptors and SARS‐CoV‐2 particles are found within both cell types in COVID‐19 patients (Ackermann et al., 2020; Carsana et al., 2020). As for the anosmia (loss of smell) experienced by COVID‐19 patients, ACE2 receptors are observed on sustentacular (support) cells in human olfactory epithelium in the nasal cavity but not on brain olfactory nerve cells in mice (Brann et al., 2020).

### 4.1 Capillary pericytes

In the heart (Chen et al., 2020; He et al., 2020) and brain (including the olfactory bulb; Brann et al., 2020; He et al., 2020) of mice, capillary *pericytes* express ACE2. Pericytes are embedded in the capillary basement membrane and are important for the formation, maintenance, and remodeling of capillaries (Armulik et al., 2011). In the lungs, severe COVID‐19 is related to an extensive loss of capillary pericytes (Burel‐Vandenbos et al., 2020), an essential substrate for angiogenesis (Kato et al., 2018) and possibly for lung repair and lung function recovery after COVID‐19. Notably, lung capillaries in COVID‐19 show extensive signs of angiogenesis, suggesting reparative processes in response to tissue hypoxia (Ackermann et al., 2020).

Some pericytes are contractile and respond to a range of vasoactive molecules (Diaz‐Flores et al., 2009). They are involved in blood flow control (Hall et al., 2014) but brain and myocardial pericytes may also die in a contracted state (“rigor”), hindering subsequent erythrocyte passage, when exposed to hypoxia and oxidative stress (Hall et al., 2014; O'Farrell et al., 2017; Yemisci et al., 2009). Pericyte damage is implicated in the development of diabetes (Richards et al., 2010), and pericyte infection could contribute to COVID‐19‐related diabetes (Coate et al., 2020). Pericytes undertake microvascular barrier functions (Armulik et al., 2010) and facilitate tissue's recruitment of immune cells in response to viral and bacterial proteins (Proebstl et al., 2012; Wang et al., 2012). During such immune responses, pericytes change phenotype to become migratory (Wang et al., 2012), possibly abandoning other functions of importance to capillary bed integrity, function, and repair.

### 4.2 Endothelial cells

In most human organs, microvascular endothelium express ACE2 surface receptors (Donoghue et al., 2000; Hamming et al., 2004), but see (He et al., 2020). SARS‐CoV‐2 particles have been observed in the endothelium of the lung (Ackermann et al., 2020), heart (Fox et al., 2020), brain (Paniz‐Mondolfi et al., 2020), skin (Colmenero et al., 2020), and kidney (Varga et al., 2020). Notably, SARS‐CoV‐2 infection of endothelial cells (ECs) is associated with changes in cell morphology and with EC apoptosis—and coincide with findings indicative of severe hypoxia in surrounding tissue. Accordingly, lung capillaries from deceased COVID‐19 patients show caliber changes as some ECs protrude into the capillary lumen (Ackermann et al., 2020), some undergo apoptosis (Varga et al., 2020), and some show signs of angiogenesis in response to severe, local tissue hypoxia (Ackermann et al., 2020). In the heart, endothelial infection is associated with EC swelling (plumpness) in small arterioles, capillaries and venules, and scattered necrosis of individual myocytes (Fox et al., 2020). In the brain, infection of subcortical white matter microvessel endothelium is associated with hyper‐acute, microscopic ischemic lesions, and older ischemic and hemorrhagic microscopic lesions (Paniz‐Mondolfi et al., 2020). Finally, EC infection in the skin is associated with endothelial swelling and in some patients with thrombosis and fibrinoid necrosis in surrounding tissue (“COVID toes”) (Colmenero et al., 2020).

Endothelial damage is likely to disturb capillary flow patterns, keeping in mind that erythrocyte diameters exceed that of the capillary lumen. Importantly, disruptions of intercellular gap junctions between endothelial cells (Ackermann et al., 2020) and endothelial cell apoptosis (above) disrupt intercellular *connexin* channels that allow signaling between endothelial cells and to upstream vascular smooth muscle cells (Isakson et al., 2006). This fast, bidirectional communication allows feed‐back control of blood flow across the microcirculation to maintain cellular oxygenation (Segal., 2005; Segal & Duling, 1986), and disruption of this communication is associated with extreme shunting of oxygenated blood through the shortest capillary pathways (Pries et al., 2010).

### 4.3 Glycocalyx

The capillary endothelium's luminal surface is covered by a 0.3–0.6 µm thick *glycocalyx* (Vink & Duling, 1996), a matrix that acts as a fluid barrier (Haaren et al., 2003) and impacts erythrocytes’ passage (Secomb et al., 1998). In critical illness, partly due to elevated TNF‐α levels, the glycocalyx is shed (Henry & Duling, 2000; Nieuwdorp et al., 2009), exposing cellular adhesions molecules that interact with immune cells to facilitate their extravasation. Cortisol *protects* the endothelial glycocalyx against TNF‐α induced shedding (Chappell et al., 2009). Glycocalyx shedding profoundly affects microvascular resistance and capillary hemodynamics (Cabrales et al., 2007; Lipowsky et al., 2011). In newly intubated COVID‐19 patients, Stahl *et al*. found evidence of glycocalyx shedding before signs of any EC injury and verified glycocalyx thinning by sublingual optical imaging (Stahl et al., 2020).

### 4.4 Capillary obstruction by neutrophils (“stalled flow”)

Neutrophils are involved in the immune response to SARS‐CoV‐2 infection. Much larger than erythrocytes and the average capillary diameter, they may occlude capillaries for several seconds, and especially when activated, cause significant capillary flow disturbances (Harris & Skalak, 1993). Neutrophil adhesion in brain capillaries impairs memory function and causes sizeable reductions in cerebral blood flow in animal models (Cruz Hernandez et al., 2019). The adhesion of hyper‐activated neutrophils to capillaries within the lungs, brain, heart, and other organs may therefore contribute to the poor prognosis of some COVID‐19 patients (Wang et al., 2020).

### 4.5 Microthrombosis

Capillary congestion and microthrombosis in the absence of upstream thrombi are common findings in the lungs of patients who died from COVID‐19‐related respiratory failure (Ackermann et al., 2020; Carsana et al., 2020). Microthrombosis has also been observed in the skin (Colmenero et al., 2020) and the kidney's glomerular capillaries (Hanley et al., 2020).

## 5 PARENCHYMAL CELL INFECTION AND/OR MICROVASCULAR DYSFUNCTION?

Capillary COVID‐19 effects may trigger symptoms and tissue injury by critically reducing local tissue oxygenation, but symptoms may also be the result of direct parenchymal cell infection.

In the lungs, infection of lung epithelium and capillary damage both seem to contribute to severe respiratory symptoms. In brain tissue, endothelial or pericyte infections, alongside systemic inflammation, may cause local hypoxia, but SARS‐CoV‐2 is also thought to be *neurotrophic* (capable of infecting nervous tissue). Nerve terminals have been speculated to take up virus particles and transmit them across synapses to other brain regions (Gu & Korteweg, 2007; Iadecola et al., 2020; Song et al., 2021). Neuropathological examination suggests that cortical neurons are infected in some patients (Song et al., 2021), possibly following entry through the olfactory mucosa (Meinhardt et al., 2020).

New‐onset diabetes in COVID‐19 patients was ascribed to direct infection of insulin‐producing pancreatic islet β cells, but recently deemed unlikely due to their lack of ACE2‐binding protein (Coate et al., 2020). ACE2 was observed on islet capillary pericytes and exocrine capillaries, however, suggesting that, in this case, a microvascular origin is more likely for the metabolic sequelae of COVID‐19 (Coate et al., 2020).

Many COVID 19 patients show elevated troponin levels early in the course of their disease, indicative of myocyte injury (Liu et al., 2020). It remains uncertain whether SARS‐CoV‐2 infects heart myocytes and cause persistent immune cell infiltration (myositis), or whether myocyte injury is the result of hypoxia, caused by pericyte infection and/or microvascular injury. Recent findings seemingly contradict the former (Fox et al., 2020). Finding the sources of heart injury in COVID‐19 remains urgent (Maleszewski et al., 2020): In one study, 78% of mostly un‐hospitalized COVID‐19 patients showed abnormal cardiac MRI 2–3 months after diagnosis (Puntmann et al., 2020).

## 6 HYPOXIA, INFLAMMATION, AND CAPILLARY FUNCTION

**FIGURE 3:**
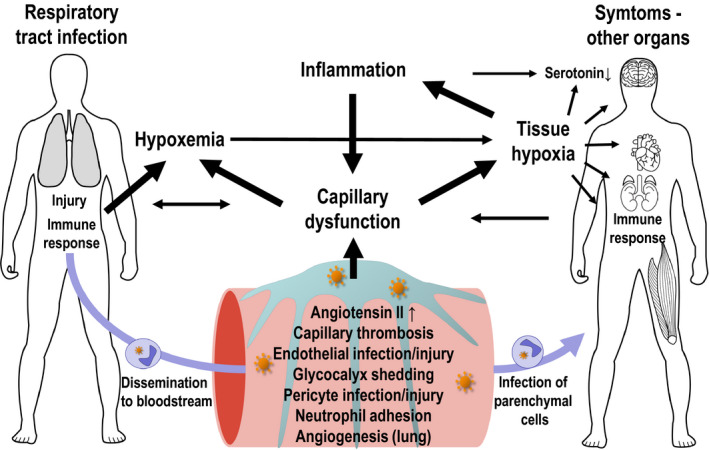
Interactions between capillary function, inflammation, hypoxia, and neurotransmission. The expression of ACE2 and other SARS‐CoV‐2 entry factors on parenchymal cells and observations of infected cells in biopsy material hold important clues to understand COVID‐19‐related organ damage

COVID‐19, like other instances of critical illness that involve systemic hypoxia (Kox et al., 2020; Sinha et al., 2020), is associated with cytokine release. TNF‐α and other inflammatory factors are known to damage the glycocalyx and impair endothelial functions (Liu et al., 2020; Zhang et al., 2020). This immune response is likely to contribute to capillary dysfunction and reduced tO_2_ in affected tissue, where cellular oxygen sensors, in turn, activate hypoxia‐inducible transcription factor (HIF) and the transcription of genes that help tissue adapt to hypoxia (Eltzschig & Carmeliet, 2011). Notably, hypoxia also activates the production of nuclear factor kappa beta (NF‐κB), the master‐switch for the transcription of genes that elicit inflammatory responses (Eltzschig & Carmeliet, 2011). Figure 3 illustrates how hypoxia‐induced inflammation, by adding further to capillary dysfunction and hypoxia, may constitute a vicious cycle in severe COVID‐19.

## 7 HYPOXIA, INFLAMMATION, AND NEUROTRANSMISSION

Low brain levels of serotonin are associated within lowered mood, anxiety, and susceptibility to depression (Praag, 2004). Oxygen is a substrate for serotonin synthesis, and even at physiological oxygen levels, oxygen availability seemingly limits 5‐HT production (Katz, 1980; Nishikawa et al., 2005). Also, when present in brain tissue, inflammatory cytokines such as TNF‐α, IL‐1, and IL‐6 give rise to *sickness behavior*—changes in subjective experience and behavior that resemble those experienced in depression (Dantzer & Kelley, 2007; Iadecola et al., 2020). Cytokines are thought to reduce serotonin levels by turning its precursor, tryptophan, into kynurenine instead. Kynurenine metabolism, in turn, reduces glutamate and dopamine release (Capuron & Miller, 2011), see also (Miller et al., 2009). These mechanisms could affect cognitive functions and quality‐of‐life for COVID‐19 patients patient with hypoxemia, high cytokine levels, and/or reduced brain tO_2_—see Figure 3.

## 8 THERAPEUTIC IMPLICATIONS?

Anticoagulant, antithrombotic, and antiplatelet drugs are already implemented in the management of COVID‐19 (Godino et al., 2020), and this review merely reiterates the need to consider microvascular function, perhaps even early in the disease. The review suggests that cellular energy crises may contribute to tissue vulnerability during severe COVID‐19. Therapies that limit metabolic demands could therefore prove protective, keeping in mind that our knowledge of COVID‐19 remains limited and that any interventions should be carefully designed, monitored, and reported. For example, maintenance of SGLT2 inhibitor treatment might be tested in diabetic COVID‐19 patients with the aim of maintaining lowered kidney oxygen demands if precautions are taken to avoid ketoacidosis (Das & Dutta, 2020). Similarly, glucocorticoids, besides protecting the microcirculation, have dose‐dependent effects on mitochondrial ATP yields that may inform optimal dosing (Du et al., 2009). The choice of sedatives during ventilator treatment may be guided by their effects on the brain's metabolic demands and blood flow, respectively (Koch et al., 2020) and monitored via plasma markers of neuronal injury and glial activation (Kanberg et al., 2020).

## 9 CONCLUSION—AND UNANSWERED QUESTIONS

SARS‐CoV‐2‐related inflammation and capillary damage may contribute synergistically to acute and long‐term COVID‐19 symptoms by interfering with blood and tissue oxygenation. Further studies of microvascular changes during COVID‐19—and especially subsequent capillary repair—are needed to understand capillary dysfunction's impact on the acute and chronic health effects of the disease. If COVID‐19‐related capillary dysfunction persists in some organs, future attention to the vascular health in younger subjects with a history of COVID‐19 may be warranted. The COVID‐19 pandemic highlights a need to develop biomarkers of capillary function across organs in humans—and may increase our understanding of the microcirculation's role in human health and disease.

## DISCLOSURES

LØ is a minority shareholder and Scientific Advisory Board member in Cercare Medical Aps, Denmark and received honoraria for lecturing from Takeda Pharmaceutical Company Limited.
